# T-Cell Infiltration and Immune Checkpoint Expression Increase in Oral Cavity Premalignant and Malignant Disorders

**DOI:** 10.3390/biomedicines10081840

**Published:** 2022-07-30

**Authors:** Subin Surendran, Usama Aboelkheir, Andrew A. Tu, William J. Magner, S. Lynn Sigurdson, Mihai Merzianu, Wesley L. Hicks, Amritha Suresh, Keith L. Kirkwood, Moni A. Kuriakose

**Affiliations:** 1 Head & Neck Surgery, Roswell Park Comprehensive Cancer Center, Buffalo, NY 14263, USA; subin.thenkunnelsurendran@roswellpark.org (S.S.); usama_aboelkheir@yahoo.com (U.A.); tu.andrew.2@gmail.com (A.A.T.); william.magner@roswellpark.org (W.J.M.); lynn.sigurdson@roswellpark.org (S.L.S.); wesley.hicks@roswellpark.org (W.L.H.J.); amritha.suresh@ms-mf.org (A.S.); 2 Pathology, Roswell Park Comprehensive Cancer Center, Buffalo, NY 14263, USA; mihai.merzianu@roswellpark.org; 3 Integrated Head and Neck Oncology Program, Mazumdar Shaw Medical Foundation Bangalore, Bangalore 560099, India; 4 Periodontics and Endodontics, University at Buffalo School of Dental Medicine, Buffalo, NY 14214, USA; klkirk@buffalo.edu

**Keywords:** oral cancer, oral potentially malignant disorders, tumor microenvironment, immunotherapy, immune cell infiltration, immune checkpoint, oncogenesis, tumorigenesis

## Abstract

The immune cell niche associated with oral dysplastic lesion progression to carcinoma is poorly understood. We identified T regulatory cells (Treg), CD8^+^ effector T cells (Teff) and immune checkpoint molecules across oral dysplastic stages of oral potentially malignant disorders (OPMD). OPMD and oral squamous cell carcinoma (OSCC) tissue sections (N = 270) were analyzed by immunohistochemistry for Treg (CD4, CD25 and FoxP3), Teff (CD8) and immune checkpoint molecules (PD-1 and PD-L1). The Treg marker staining intensity correlated significantly (*p* < 0.01) with presence of higher dysplasia grade and invasive cancer. These data suggest that Treg infiltration is relatively early in dysplasia and may be associated with disease progression. The presence of CD8^+^ effector T cells and the immune checkpoint markers PD-1 and PD-L1 were also associated with oral cancer progression (*p* < 0.01). These observations indicate the induction of an adaptive immune response with similar Treg and Teff recruitment timing and, potentially, the early induction of exhaustion. FoxP3 and PD-L1 levels were closely correlated with CD8 levels (*p* < 0.01). These data indicate the presence of reinforcing mechanisms contributing to the immune suppressive niche in high-risk OPMD and in OSCC. The presence of an adaptive immune response and T-cell exhaustion suggest that an effective immune response may be reactivated with targeted interventions coupled with immune checkpoint inhibition.

## 1. Introduction

Oral cavity potentially malignant disorders (OPMD), such as leukoplakia and erythroplakia, are often the precursors to invasive squamous cell carcinoma of the oral cavity. Oral squamous cell carcinoma (OSCC) accounts for more than 300,000 cases each year worldwide [1] with a static 5-year survival rate of <50% [2,3]. The pathology of these oral cavity disorders may exhibit progression from hyperplasia and dysplasia to frank invasive cancer [4]. Dysplasia is pathologically divided into mild, moderate and severe. It is extremely difficult to clinically distinguish which of these lesions will progress to malignancy. Furthermore, there is a scarcity of literature addressing the underlying mechanisms, particularly those of the tumor microenvironment (TME) in disease progression.

Characterizing tumor niche, especially the immune cells, during early carcinogenesis will enable a heightened understanding of the cell types and their interactions during the early events of tumor immune evasion. We are interested in understanding how the TME contributes to oral carcinogenesis. While the oral carcinoma immune profile is well-characterized [5,6], comparatively few studies have addressed the role of the immune cell niche in premalignant lesions [7,8].

Two publications have described the accumulation of regulatory T cell (Treg) and immune checkpoint expression in the oral cancer TME; however, they only compared OSCC to normal tissue [6,9]. Immune checkpoints dampen adaptive immune responses and contribute to tumor evasion. A single publication demonstrated Treg accumulation in OPMD and OSCC [8]. Our previous work examined CXCL12 and CXCR4, a chemokine axis known to recruit Treg, in OPMD and OSCC [10].

Two additional publications addressed PD-1 and T cells in limited oral leukoplakia cohorts without precise dysplasia classification [11,12]. Our aim was to address gaps in the literature through determination of Treg, CD8 and immune checkpoint levels during progression to oral cancer. In the current study, we postulate that there are specific changes in the OPMD TME that are associated with the progression of these lesions to carcinoma.

We hypothesize that regulatory T cells and immune checkpoint molecules accumulate in the OPMD TME and correlate with oral dysplastic progression to OSCC. To test this hypothesis, we investigated Treg (CD4, CD25 and FoxP3), Teff (CD8) and immune checkpoint molecule (PD-1 and PD-L1) expression across the spectrum of oral dysplasia (mild, moderate and severe) to invasive carcinoma. Our study of OPMD and its TME immune components may be an important step towards identifying pathways of tumor malignancy and immune escape.

## 2. Materials and Methods

### 2.1. Patient Cohorts

The study was approved by the Institutional Review Board (RPCI IRB No: I66805) and conducted on de-identified formalin-fixed paraffin-embedded (FFPE) oral lesion samples from patients with histologically confirmed oral potentially malignant disorders (OPMD) or oral cancer (OSCC) who presented to the Department of Head and Neck Surgery, Roswell Park Comprehensive Cancer Center (2006 to 2013). Biopsies with clinically and histologically normal mucosa were used as controls. Samples were histologically classified as normal mucosa; parakeratosis/hyperplasia; mild, moderate or severe dysplasia; and carcinoma based on pathology assessments. An ordinal score between 1 and 6 was assigned to stratify the lesions as normal tissues (1), parakeratosis/hyperplasia (2), mild (3), moderate (4), severe dysplasia (5) and carcinoma (6).

### 2.2. Immunohistochemical Staining

FFPE blocks were sectioned (5 µm) for slides and then deparaffinized sequentially in xylene and ethanol. Antigen retrieval was performed in TRS (Target Retrieval Solution, High pH, Dako, Denmark) for 30 min steaming followed by 20 min at room temperature. The slides were incubated with 0.3% hydrogen peroxide in methanol to block endogenous peroxidases, rinsed with tris-buffered saline (TBS, Dako, Denmark) and incubated overnight at 4 °C with primary antibodies (Supplementary Table S1). Antibody detection was performed using mouse- and rabbit-specific HRP/DAB (ABC) secondary antibody detection kits (Abcam-ab64264) following the manufacturer’s protocol. All slides were counterstained with Mayer’s hematoxylin, mounted and scanned using (Aperio ScanScope XT 1509, AT2, Leica Biosystems, IL, USA) at 20(×) magnification.

### 2.3. Scoring and Analysis of Immunohistochemical Data

The presence of tumor-infiltrating lymphocytes (TIL) was quantitatively scored by counting the cells positively stained for FoxP3, CD4, CD8, CD25 and PD-1 [6,13]. An independent pathologist defined the area of sections to be assessed based on histology, and two independent reviewers blinded to clinicopathological data of the samples assessed the staining of each slide. Each image from the Aperio Scanscope (20(×) magnification) was raised 20(×) in Imagescope analysis software (Leica biosystems, IL, USA) resulting in a high-power field (hpf) with a total magnification of 400(×). The number of positively stained cells was counted in each hpf until the entire section was evaluated. In every section, 3–15 hpfs were scored and averaged to obtain the final count. The values from both reviewers were averaged, and discrepancies in scoring were re-assessed by both reviewers.

The expression of PD-L1 was quantitated using the H-score method. The sum of the intensity (0 = negative staining; 1+ = weak staining; 2+ = moderate staining; and 3+ = strong staining) and percentage of positively stained tumor cells was calculated with the H-score ranging from 0 (negative) to 300: H-score = (1 × % weakly stained cells) + (2 × % moderately stained cells) + (3 × % strongly stained cells) [13].

### 2.4. Statistical Analysis

All statistical analyses were performed using IBM SPSS Statistics v26 (IBM, Armonk, NY, USA), including standard frequency and descriptive assessments as well as bivariate correlation analysis and multivariate analysis. Pearson (scale) and Spearman (ordinal) correlations were analyzed as applicable. Significance was determined with the Chi-square test (two-tailed sigma <0.05). Survival analysis was performed using the Kaplan–Meier algorithm with significance determined by the Mantel–Cox Log Rank test *p* < 0.05. Power analyses were performed in G*Power 3.1.

## 3. Results

### 3.1. Patient Characteristics

A cohort of 270 OPMD and malignant tissue samples obtained from 128 patients was evaluated for CD4, CD8, FoxP3, CD25, PD-1 and PD-L1. Patient demographics and smoking status are described in Table 1. Lesions with mild or no dysplasia were classified as low-risk, while moderate dysplasia, severe dysplasia and carcinoma in situ were considered high-risk lesions (Table 2). The malignant lesions included micro-invasive carcinoma and carcinoma regardless of stage and differentiation.

### 3.2. Immune Marker Detection across Oral Cancer Stages

**Figure 1:**
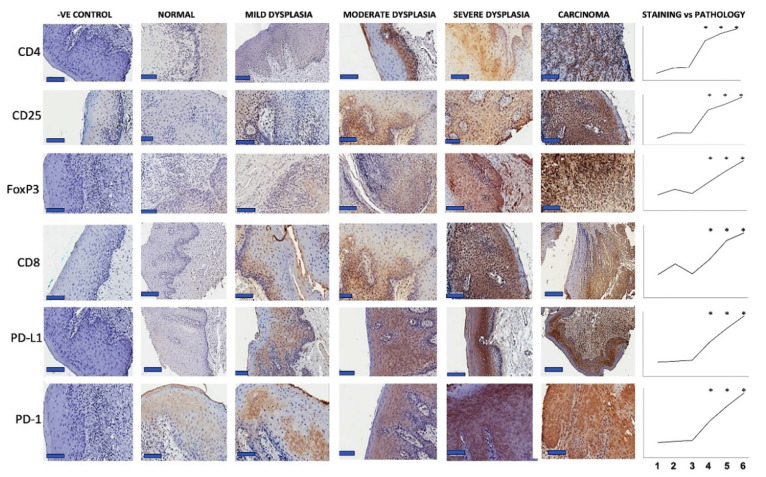
Infiltrating lymphocytes and immune checkpoint expression levels increase in parallel with oral cancer progression. Immunohistochemical analysis of immune markers in patient clinical samples pathologically defined as normal mucosa, pro-gressive grades of dysplasia (mild, moderate, and severe dysplasia) and carcinoma. Representative images are shown at total magnification 400(×) (scale bar, 100 µm). Average IHC staining for each immune marker was calculated and correlated with pa-thology (ordinal scale). The Y-axis in the graph indicates the IHC score and the X-axis indicates the pathological distribution of patient cohorts 1-normal, 2- parakeratosis (images not shown), 3-mild dysplasia, 4-moderate dysplasia, 5-severe dysplasia and 6-carcinoma. Pathology cohorts whose IHC staining value is significantly different from the normal group are indicated (*, *p* < 0.05).

The first goal of our study was to identify the presence of Teff, Treg and PD-1/PD-L1 in OPMD and OSCC microenvironments. To quantify the presence of regulatory T cells, we performed immunohistochemical staining for CD4, CD25 and FoxP3 on the FFPE sections (Figure 1) (Table 3). We also stained for effector T cell marker CD8 and immune checkpoint components PD-1 and PD-L1 in these same samples. Increasing the expression of all these markers was evident throughout disease progression as reflected by the average number of positively stained cells for immune markers and PD-1 and H-score for PD-L1. (Figure 1) (Table 3). Correlation coefficients were determined between the IHC scores and ordinal designations of pathology (Figure 1) (Table 4). The Spearman correlations suggest that the levels of each of these markers in the oral microenvironment increase with disease progression.

The intensity of CD25 staining correlated significantly (*p* < 0.01) with the severity of dysplasia (Figure 1) (Table 4). A similar pattern was observed for CD4 wherein high-risk dysplastic and carcinoma samples showed higher infiltration compared to normal tissues (*p* < 0.01) (Figure 1) (Table 4). The presence of FoxP3^+^ cells strongly correlated with pathology (*p* < 0.01) (Figure 1) (Table 4). As Tregs are expected to express CD4, CD25 and FoxP3, we tested the correlations between these markers (Table 4). The strong positive correlations between CD4, CD25 and FoxP3 (correlation coefficients 0.64–0.96, *p* < 0.01) support our hypothesis that Tregs are recruited to OPMD and OSCC. Increased Treg levels with advanced pathology indicate their probable role in immune escape and potential role in immunotherapy resistance.

CD8^+^ T cells are the effector T cells critical to immune-mediated tumor control and to immunotherapy responsiveness. To quantify Teff infiltration into OPMD and oral cancer lesions, we performed CD8 immunohistochemistry on our panel of oral tissues. Our results identified cytotoxic T cells within the oral tissues and OSCC TME (Figure 1) (Table 3), and the presence of CD8^+^ cells positively correlated with dysplastic progression (Spearman coeff = 0.49, *p* < 0.01) (Table 4).

It has been previously demonstrated that activated effector T cells can be inhibited in the TME by tumor expression of PD-1 ligands [14,15,16]. To determine the expression of this immune checkpoint in OPMD and OSCC, we stained our oral tissue panel for PD-1 and PD-L1 (Figure 1) (Table 3). PD-L1 IHC scores in each OPMD/OSCC category were higher than the normal samples and reached statistical significance at moderate dysplasia. This increase in PD-L1 staining associated with dysplastic progression was validated by its Spearman correlation coefficient (0.52 *p* < 0.01) (Table 4).

Similarly, PD-1 detection began to increase in the mild dysplasia samples and was significantly elevated in moderate and severe dysplasia as well as carcinoma samples (Spearman correlation coefficient 0.54, *p* < 0.01) (Table 4). Importantly, our PD-1 and CD8 quantitation closely correlated with each other (*p* < 0.001) suggesting the expected colocalization. Similarly, CD4 cells were also tightly correlated with PD-1 expression. PD-1 detection did not correlate with CXCL12, which is expressed by stromal cells, including cancer-associated fibroblasts supporting the specificity of our IHC assessment.

### 3.3. Correlations between Treg, Teff and Immune Checkpoints

Our analysis demonstrated that Treg and Teff were present at multiple stages of oral mucosal dysplasia and cancer. It is possible that these populations play a mechanistic role in tumor development, as these patterns appear to be strikingly similar. To quantify these patterns, we examined the Pearson correlation coefficients between CD8 and Treg IHC values (CD8:FoxP3 correlation coefficient 0.54, *p* < 0.01) (Table 4). The close alignment of these populations may indicate a distinct mechanism for suppressing effective CD8^+^ cytotoxic T lymphocyte (CTL)/Teff responses in the oral TME.

The PD-L1 levels were also closely correlated with CD8 levels (Pearson correlation coefficient 0.51, *p* < 0.01) (Table 4) suggesting that, although Teff were present in the lesion and tumor microenvironments, they could be subject to immune checkpoint inhibition and rendered ineffective due to exhaustion.

### 3.4. CXCR4-CXCL12 Correlation with Immune Landscape

Our previously published work in OPMD that dealt with cancer stem cells and markers of chemokine pathways indicated that the presence of CXCL12 (SDF-1) and CXCR4 in oral premalignant and malignant sections correlated with disease progression [10]. The patient samples described here were included in our previous cohorts. The CXCL12:CXCR4 axis is known to facilitate homing of Treg; thus, we incorporated these published IHC data into our new analysis. CXCR4 cytoplasmic staining was positively correlated with CD25 (Treg) as well as the immune checkpoint marker PD-1 (*p* < 0.05) (Table 4).

### 3.5. Survival Analysis

**Figure 2:**
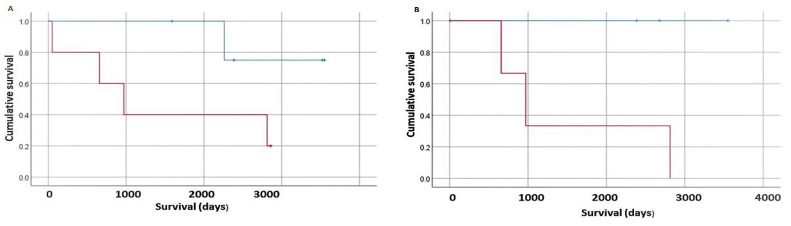
**Survival analysis based on Treg surrogate markers.** (**A**), Kaplan–Meier analysis of OPMD/OSCC patients stratified by first vs. fourth quartile of CD25 staining. The red line represents the highest quartile CD25 detected, and the blue line represents the lowest quartile of CD25 detection. The survival difference did not reach statistical significance—Mantel–Cox Log Rank test *p* = 0.092. (**B**) Kaplan–Meier analysis of OPMD/OSCC patients stratified by first vs. fourth quartile of FoxP3 staining. The red line represents the highest quartile FoxP3 detected, and the blue line represents the lowest quartile of FoxP3 detection. The survival difference did not reach statistical significance—Mantel–Cox Log Rank test *p* = 0.063.

In a small subset of OSCC patients, survival analysis was performed. IHC scores for each marker were analyzed based on quartiles and survival analyses were performed for the highest vs. lowest staining levels. Since few patients in this study had overall survival data available, the date of last follow up was considered as a surrogate marker (N = 19). Kaplan–Meier survival analyses revealed intriguing trends suggesting that lower levels of Treg recruitment (CD25 and FoxP3) were associated with improved survival; however, none reached statistical significance (Figure 2).

## 4. Discussion

Immune dysfunction within the OPMD and OSCC TME may contribute to disease evolution [17,18,19]. Immune changes during dysplastic progression have not been well defined. Therefore, a better understanding of these immune landscapes would provide insights into underlying mechanisms and potential therapeutic targets. The TME includes both pro-tumorigenic and anti-tumorigenic immune cells. The regulation and balance of these cell populations influence the immune responses within the tumor cell/stromal framework, including mechanisms that support escape from immune surveillance [20,21] and subsequent malignant transformation.

Although the oral carcinoma immune profile is well characterized [5,6], few studies have addressed the role of the immune cell niche during oral carcinogenesis. Kouketsu et al. demonstrated Treg accumulation in OPMD and OSCC [8]. Chen et al. reported CD8 and PD-L1 increased expression in OSCC versus healthy tissues [12]. Their samples included leukoplakia, of which six samples were considered dysplastic.

We confirmed and extended these observations by incorporating chemokine pathway, immune checkpoint and Teff infiltration detection in a cohort of patient samples that included normal, mild, moderate and severe dysplasia as well as carcinoma. The data reported here is the first thorough examination of CD4, CD8 and immune checkpoint changes through pathologically defined stages of oral dysplasia. Where data were available, we analyzed the impact of these immune microenvironment changes on patient survival.

Lymphocyte infiltration into the TME is generally viewed as an indication of host immunity against the tumor, and its presence may alter tumor biology [22,23]. CD8^+^ Teff (CTL) are immune anti-tumor effector cells whereas regulatory T cells (Treg, CD25^+^CD4^+^FoxP3^+^) can suppress this cytotoxic T-cell function. The interplay between Treg and Teff is crucial in immune evasion by cancer cells, anti-tumor response and immune homeostasis. The presence of CD8^+^ T cells is associated with a more favorable clinical prognosis [24,25]. However, Treg express inhibitory coreceptors and release immune-suppressive cytokines, thereby, hampering cytotoxic T lymphocytes, natural killer (NK) cells, dendritic cells (DC) and B cell function as well as dampening anti-tumor immunity [26,27,28,29,30].

Treg cells express the chemokine receptor CXCR4 and can migrate along a gradient of its ligand CXCL12 [31]. This axis can contribute to Treg recruitment to the TME and the resulting immune-suppressive tumor environment. Our previously published work demonstrated the enhanced expression of CXCL12 and CXCR4 during malignant progression of oral dysplastic lesions [10]. In aggregate, the data presented by this and other studies reflect the importance of both the immune response and epithelial stromal interactions in the regulation/progression of disease.

This study quantified the presence of specific T cell subsets (CD4, CD25, FoxP3 and CD8) in the stromal milieu along with activation of immune modulator pathways during OPMD progression and in OSCC. Our results identified the presence of an increasingly immunosuppressive microenvironment during dysplastic progression as indicated by Treg recruitment and activation of the PD-1 immune checkpoint axis. This immune shift correlated with increases in CXCR4, CXCL12 and CD44 shown in our previous work [10]. CD8^+^ T cell recruitment in early lesions increased along the spectrum of progression and in OSCC.

The presence of CD8^+^ T cells in dysplastic lesions and OSCC may be a positive prognostic indication; however, the coordinate induction of immune checkpoint expression suggested an additional layer of immune suppression in oral dysplasia and carcinoma. The joint recruitment of Treg and Teff as well as the induction of immune checkpoint expression establish the balance between a productive immune response and immunotherapy resistance. Understanding this balance will provide novel targets for therapeutic intervention to improve patient responses. The identification of these features in dysplastic lesions may support earlier intervention that might disrupt this reinforced suppressive environment.

Several studies have shown strong tumor infiltration by CD8^+^ cells in head and neck cancer [32,33]. A tongue cancer study indicated that malignant transformation was accompanied by an increase in CD4^+^ and B cells, in addition to CD8^+^ and CD14^+^ cells [18]. We focused on CD4^+^ Treg, CD8^+^ Teff cells and the PD-1 immune checkpoint and their potential association with pre-malignant and malignant oral cavity lesions. The data reported here revealed a strong correlation between CD4, CD25 and FoxP3 markers, indicating a high incidence of Treg cells, which in turn correlated with dysplastic progression to carcinoma. We also found significantly increased numbers of CD8^+^ cells with increasing severity of dysplasia in our patient cohorts, which is consistent with studies performed on other malignancies [34,35,36,37].

Recent studies emphasized the role of CD8^+^ T cells in the control of tumor growth and the prolongation of patient survival [35,38]. The concurrent presence of Treg and CD8^+^ T cells in our study, however, suggests a potentially responsive immune environment that has shifted to immunosuppression. Additionally, the significant drop in the CD8/Treg infiltration in the high-risk group as compared to the low-risk cohort suggested that the low-risk cohort (mild to moderate dysplasia) might benefit from immunological modulation.

The correlation between FoxP3 and disease grade indicated an increase in Treg recruitment to oral cavity lesions during disease progression. Since Treg can counteract the effect of immune checkpoint blockade, their presence could impair immune attack and result in decreased survival. The limited patient follow up in our dataset revealed a trend toward Treg recruitment having a negative impact on survival. Future studies with more extensive patient observation will strengthen this association.

Immune checkpoints are critical regulators of immune homeostasis, have been co-opted by tumors to support immune evasion and are now therapeutic targets showing variable disease site efficacy. PD-L1 overexpression in solid tumors, including head and neck cancers [39], can provide direct tumor protection and reduce the activity of PD-1 expressing tumor-infiltrating effector CD8^+^ T cells [40,41]. PD-L1 expression in oral dysplasia has not been well characterized.

Our study indicated that the PD-1/PD-L1 axis correlated with disease progression. The combined increase in the expression of PD-1/PD-L1 and Treg suggested a highly immune suppressive environment in this cohort. Although ICI immunotherapy has been approved for head and neck squamous cell carcinoma, a clinical response is only observed in <20% of patients [42,43]. The primary challenges in checkpoint inhibitor therapy are the overlapping, parallel and synergistic immune escape pathways that tumor cells utilize to modulate the niche and survive immune surveillance and therapy.

Our data describe Treg, Teff and immune checkpoint changes across pathologically defined stages of dysplasia and provide a longitudinal view of the immune microenvironment during OPMD/OSCC progression. However, our approach cannot address every marker or cell type of interest. Future studies using high-throughput methods will be necessary for complete characterization of the dysplastic immune microenvironments. Interest has recently been expressed in the potential use of immunotherapy to prevent progression of dysplastic lesions [44]. Further characterization of these pathways and their modulators in dysplasia and OSCC is likely to identify novel targets for both chemoprevention and chemotherapy.

## 5. Conclusions

Our results established a combined presence of Treg and CD8^+^ T cells and immune checkpoint molecule expression in OPMD/OSCC microenvironments. Our data demonstrated dynamic changes in the numbers and balance among these immune components across the spectrum of dysplastic progression and carcinoma. These data support the feasibility of immunotherapy for OPMD and its potential application in OSCC chemoprevention.

Our results also suggest that T-cell exhaustion may contribute to immune escape and immunotherapy resistance in dysplastic progression to OSCC. These data highlight the need for the parallel targeting of mechanisms contributing to the immune suppressive niche in high-risk OPMD and in OSCC. The accumulation of Treg, as well as CD8^+^ Teff relatively early in the dysplastic progression suggests that a combinatorial intervention, including immune checkpoint inhibition, may prevent progression by interrupting immune escape or enhancing the immunotherapeutic response.

## Figures and Tables

**Table 1:** Patient demographics.

Characteristic	Cases	Percentage
Sex		
Male	71	55.5%
Female	57	45.5%
**Age Group (years)**		
28–50	30	23.4%
51–60	40	31.3%
61–70	35	27.3%
71+	23	18.0%
**Race**		
White	116	90.6%
Black	6	4.7%
Asian	3	2.3%
American Indian or Alaskan Native	1	0.8%
Hispanic	1	0.8%
Other	1	0.8%
**Ever Smoker**		
Yes	82	64.1%
No	45	35.9%
**Total Patients**	128	100%
**Mean Age = 59.9, Median 60.0**		

**Table 2:** Pathology distribution of samples.

**Pathology**	**N**	**Percentage**
Normal	87	32.2%
Hyperplasia/Parakeratosis	45	16.7%
Mild Dysplasia	69	25.6%
Moderate Dysplasia	22	8.1%
Severe Dysplasia/Carcinoma in situ	13	4.8%
Carcinoma	34	12.6%
**Total Samples**	**270**	

**Table 3:** T cell, checkpoint molecule and chemokine quantitation in oral potentially malignant and malignant lesions.

Pathology		CD25 (N:247)	CD4 (N:263)	FoxP3 (N:248)	CD8 (N:247)	PD-L1 (N:256)	PD-1 (N:253)	CXCR4 ^ (N:209)	CXCL12 ^ (N:211)
Normal	Mean ^#^	15.6	17.0	39.3	25.2	34.3	32.0	2.7	115.0
	SEM	1.7	1.6	3.5	1.9	5.3	5.3	0.3	6.6
Parakeratosis	Mean	31.4	32.0	54.4	37.0	36.4	25.5	2.3	112.8
	SEM	6.7	5.1	6.8	4.0	7.7	6.0	0.4	10.2
Mild dysplasia	Mean	28.5	31.0	43.0	26.0	39.0	38.6	3.2	127.7
	SEM	3.8	2.7	4.0	2.1	4.6	4.5	0.3	6.9
Moderate dysplasia	Mean	83.3	92.3	72.2	41.6	82.1	97.0	3.4	93.6
	SEM	11.0	11.8	12.8	5.5	13.8	14.7	0.7	22.7
Severe dysplasia	Mean	114.1	129.1	101.1	69.5	127.3	112.5	5.0	198.1
	SEM	12.8	13.2	12.8	7.9	17.0	20.6	1.0	20.4
Carcinoma	Mean	103.2	109.4	120.4	65.1	134.3	139.3	5.0	153.5
	SEM	9.6	8.7	6.3	5.8	14.1	16.2	0.7	16.4

^, Published data [10]; ^#^, average number of positively stained cells/hpf (CD25, CD4, FoxP3, CD8, PD-1, CXCR4), average Histoscore/hpf (PD-L1, CXCL12); and SEM, Standard Error of the Mean.

**Table 4:** Correlations between pathology and IHC assessments.

	Pathology	CD25	CD4	FoxP3	CD8	PD-L1	PD-1	CXCR4 ^	CXCL12 ^
Pathology	1.00								
CD25	**0.64**	1.00							
CD4	**0.70**	**0.96**	1.00						
FoxP3	**0.52**	**0.71**	**0.69**	1.00					
CD8	**0.49**	**0.79**	**0.78**	**0.54**	1.00				
PD-L1	**0.52**	**0.54**	**0.58**	**0.48**	**0.51**	1.00			
PD-1	**0.54**	**0.53**	**0.55**	**0.45**	**0.46**	**0.74**	1.00		
CXCR4 ^	0.27	0.17 ^#^	0.23	0.14	0.22	0.35	0.19 ^#^	1.00	
CXCL12^	0.20	0.14 ^#^	0.17	0.15	0.12	0.32	0.29	0.32	1.00

IHC vs. pathology (scored on an ordinal scale) associations are presented as Spearman correlation coefficients whereas correlations between markers are presented as Pearson correlation coefficients. bold, *p* < 0.01; ^#^, *p* < 0.05; and ^ published IHC data re-analyzed in the context of new data presented here [10].

## Data Availability

The data presented in this study are available on request from the corresponding author.

## Supplementary Materials

The following supporting information can be downloaded at: https://www.mdpi.com/article/10.3390/biomedicines10081840/s1.
