# Supplementary material for: T-Cell Infiltration and Immune Checkpoint Expression Increase in Oral Cavity Premalignant and Malignant Disorders
Source: Biomedicines. 2022 Jul 30;10(8):1840. doi: 10.3390/biomedicines10081840 (PMC9404942; doi:10.3390/biomedicines10081840)
Supplement: Supplementary file 1 [file biomedicines-10-01840-s001.zip › biomedicines-1820481-supplementary.pdf]

Supplementary Materials: Table S1

Ab Name	Manufacture	Host species	Reacts with	Dilution
CD25	Epitomics	Rabbit monoclonal	Human	1:100
CD4	Dako	Mouse monoclonal	Human	1:60
FoxP3	Abcam	Mouse monoclonal	Human	1:100
CD8	Abcam	Mouse monoclonal	Human	1:200
PD-1	Abcam	Mouse monoclonal	Human	1:100
PD-L1	Abcam	Rabbit monoclonal	Human	1:100
